# Utilizing Multiple Triboelectric Nanogenerator Sensors and Signal Processing Technology for Monitoring Periodic Leg Movements of Sleep

**DOI:** 10.3390/bios14110532

**Published:** 2024-11-04

**Authors:** Zongyi Jiang, Yunzhong Wang, Damian Tohl, Liming Fang, Youhong Tang

**Affiliations:** 1 Medical Device Research Institute, College of Science and Engineering, Flinders University, Adelaide, SA 5042, Australia; jian0393@flinders.edu.au (Z.J.); steven.wang@flinders.edu.au (Y.W.); damian.tohl@flinders.edu.au (D.T.); 2 National Engineering Research Centre for Tissue Restoration and Reconstruction, South China University of Technology, Guangzhou Higher Education Mega Centre, Panyu District, Guangzhou 510006, China; lmfang@scut.edu.cn

**Keywords:** periodic leg movements of sleep, triboelectric nanogenerator, biosensors, multi-sensing, home-based sleep monitoring

## Abstract

High-quality sleep is essential for both physiological and cognitive functions. However, periodic leg movements of sleep (PLMS), an involuntary phenomenon during sleep, affects millions of people worldwide, contributing to sleep fragmentation and functional impairments. The accurate monitoring of PLMS is important for identifying and addressing these issues. Traditional methods, such as polysomnography (PSG), which monitor the bare tibialis muscle movements in clinical environments, may not adequately reflect the natural sleep patterns at home. They are costly and unsuitable for long-term studies. In recent years, there has been growing interest in using flexible sensors for sleep monitoring. Previous studies have applied triboelectric nanogenerators (TENGs) as flexible sensors to detect muscle movements during sleep. However, distinguishing true PLMS from false signals caused by external factors, such as blankets, remains a challenge. This study proposes a method using three TENG sensors placed on the dorsum, ankle, and tibialis, respectively, along with signal processing techniques to enhance the accuracy of PLMS detection. This study provides a cost-effective, comfortable method for PLMS monitoring, with the potential for widespread use in home-based sleep studies and long-term care in the future.

## 1. Introduction

Periodic leg movements of sleep (PLMS) are involuntary movements that occur during sleep, characterized by repetitive and stereotyped limb motions that recur in episodes [1,2]. Symonds (1953) referred to PLMS as “nocturnal myoclonus”, interpreting these movements as a type of nocturnal epilepsy [3]. In 2006, the World Association of Sleep Medicine (WASM) established guidelines for recording and scoring PLMS. Traditional polysomnography (PSG) detects PLMS using surface electrodes positioned longitudinally and symmetrically around the midsection of the tibialis anterior muscle [4]. The leg movement (LM) should be scored as increased electromyogram (EMG) activity lasting between 0.5 and 10 s, exceeding 25% of the baseline amplitude. PLMS is defined as the consecutive sequence of four or more LM whose inter-movement intervals are between 5 and 90 s [5]. The severity of PLMS is usually quantified with the PLMS index, which indicates the number of PLMS per hour of recording, and is considered abnormal when it exceeds the value of 15 in adults [4]. When assessing PLMS patients, it is essential to monitor their sleep over multiple nights, as symptoms can vary from night to night [6,7].

In PLMS, the movements are not limited to the tibialis anterior muscle alone. Typical PLMS movements include dorsiflexion of the ankle and extension of the big toe [8]. The tibialis anterior often works in coordination with the gastrocnemius and soleus muscles to produce dorsiflexion or plantar flexion of the foot. The hallux (big toe) usually moves in sync with other parts of the foot, especially during dorsiflexion or plantar flexion. Ankle movements are also commonly synchronized with the activity of the tibialis anterior, gastrocnemius, and soleus, with these muscle groups contributing to the corresponding dorsiflexion or plantar flexion of the ankle [9]. Previous research by Bobovych et al. has verified that, in addition to the tibialis anterior, the dorsum and ankle of the foot are also effective locations for sensor placement in the detection of PLMS [10,11].

Over the past few years, there has been growing interest in triboelectric nanogenerator (TENG) as a new approach to sleep monitoring. According to research, the cost of a single night’s polysomnography (PSG) can range from USD 600 to USD 1100, placing a financial strain on patients [12]. TENG offers a costly and potentially more comfortable alternative. It allows for less disruptive sleep monitoring to be performed at home. TENG-based sensors may address these weaknesses by offering detailed movement detection through multiple sensors placed on different body parts, self-powering capabilities from body movements, high sensitivity to small movements, enhanced signal quality through signal processing techniques, and reduced environmental interference [13]. Ding et al. created a sleep monitoring belt that incorporates a CNT-doped porous TENG. This belt features two TENG components operating in parallel, each with its own independent signal pathways, and is positioned beneath the chest to capture breathing and heartbeat signals during sleep [14]. Meanwhile, Kou et al. designed a smart pillow utilizing a flexible sensor to track head movements throughout the night. This pillow employs porous polydimethylsiloxane (PDMS) and TENG pressure-sensing arrays to map pressure distribution and monitor motion trajectories, specifically focusing on head movements and body repositioning during sleep [15]. The lightweight and conformable nature of these flexible sensors allows for direct contact with the skin, offering more accurate monitoring capabilities and making them a potentially more user-friendly solution for long-term sleep monitoring in a home setting [13].

**Figure 1:**
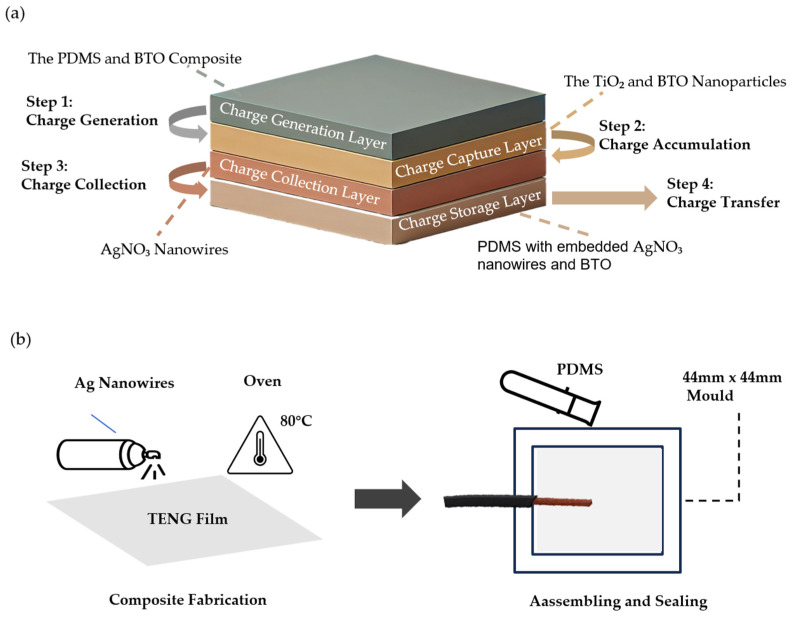
(**a**) Schematic diagram showing the charge generation, capture, collection, and storage processes of the composite multi-material triboelectric nanogenerator (TENG) used in this study, forming a complete energy cycle. (**b**) Composite fabrication: Spin-coated Ag nanowires on TENG film, cured at 80 °C for 20 min. Assembly and sealing: The composite and a conducting wire were placed in a box and sealed with PDMS. TENG sensor prototype: Length of 44 mm, width of 44 mm, height of 2 mm.

The contact and separation mode of a TENG uses the principles of triboelectrification and electrostatic induction to generate electricity through the contact and separation of materials. When these materials come into contact, charge separation occurs at the interface, generating an electrical signal. When the materials in the TENG separate, the charges redistribute, leading to the generation of another electrical signal [16]. The TENG films used in this study were provided by Professor Liming Fang from South China University, where they were previously validated as effective sensors in his earlier research [17]. The composite multi-material triboelectric nanogenerator (TENG) is featured with multiple active layers, with each layer contributing to charge generation, capture, and storage. Figure 1a illustrates each layer’s contribution and the operational processes of TENG used in this study. The gray layer serves as the charge generation layer, composed of a composite material of PDMS and barium titanate (BTO), which acts as the primary triboelectric layer that generates charges through friction and separation with the skin surface. In detail, step 1 shows the generation of charges resulting from the contact and friction between the composite layer and the skin. The champagne-colored layer represents the charge capture layer, where titanium dioxide (TiO_2_) and BTO nanoparticles act as charge capture elements, effectively capturing and retaining the generated charges to prevent rapid dissipation. Step 2 is the accumulation of charges. The brown layer functions as the charge collection layer, with embedded silver nitrate (AgNO_3_) nanowires serving as conductive pathways that efficiently collect and transfer charges. Step 3 demonstrates the collection of charges, showing how AgNO_3_ nanowires conduct the accumulated charges toward a connected electrode or external circuit. The light brown layer represents the charge storage layer, where the entire composite structure (PDMS with embedded AgNO_3_ nanowires and BTO) may also act as a temporary storage area before the charges are conducted away. Step 4 illustrates the charge transfer, displaying the charges being transferred out from the TENG for external use, thus completing the cycle and readying it for the next contact–separation event. The TENG film underwent reprocessing to better suit the needs of this experiment. A schematic diagram illustrating the processing of the TENG sensor prototype is shown in Figure 1b. To enhance its conductivity, AgNO_3_ nanowires were spin-coated onto the TENG film and cured at 80 °C for 20 min. Subsequently, the composite material and a conductive wire were placed inside a 44 mm × 44 mm mold and sealed with PDMS to improve the moisture resistance.

In natural sleep conditions, external factors, such as blanket friction, one foot resting on top of the other, or turning the foot left and right, will affect the monitoring activity, making it challenging to accurately identify true PLMS events. To address this, our study employed three TENG sensors strategically placed on the dorsum, ankle, and tibialis anterior muscle, respectively. By monitoring these effective positions simultaneously, the combined data from the sensors could be analyzed to differentiate genuine PLMS from false signals. This study implemented signal processing techniques to classify the signals obtained from the three sensors under various environmental conditions. By doing so, we aimed to enhance the accuracy of PLMS detection, reduce false positives caused by external factors, and improve the reliability of long-term, home-based sleep monitoring.

## 2. Methods and Evaluation

### 2.1. Measurement Setup

**Figure 2:**
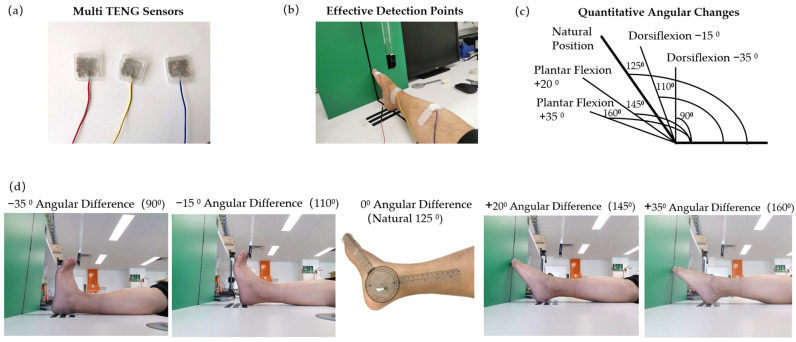
(**a**) The TENG sensor prototypes used in this study. These TENG sensors were validated in previous research [17]. Prior to conducting this experiment, each of the three sensors underwent signal detection tests to ensure their reliability. (**b**) The first sensor was attached to the dorsum of each foot at the base of the big toe using tape, the second sensor was placed on the ankle, and the third sensor was fixed to the middle of the anterior tibialis muscle according to suggestions from previous research [4,10,11]. (**c**). The graph illustrates the four angular changes, starting from a natural resting angle of 125 degrees. The angle then shifted to 90 degrees, followed by 110 degrees, 145 degrees, and finally 160 degrees. Each point on the graph represents a transition between these specific angles, showing the dynamic range of motion observed during the study. (**d**) Four movement angles were determined by fixing the heel at a set distance from the wall.

Figure 2a shows the TENG sensors used in this study. Figure 2b shows where the three TENG sensors were placed on the dorsum, ankle, and tibialis anterior of a male participant right leg to monitor PLMS [4,10,11]. In a neutral position, the participant’s foot formed an angle of 125° with the leg. PLMS were simulated by performing dorsiflexion at two fixed angles (90° and 110°) and plantar flexion at two fixed angles (145° and 160°), which are shown in Figure 2c,d. All measurements were conducted under indoor conditions with a relative humidity of 46%.

### 2.2. Hardware System Design

**Figure 3:**
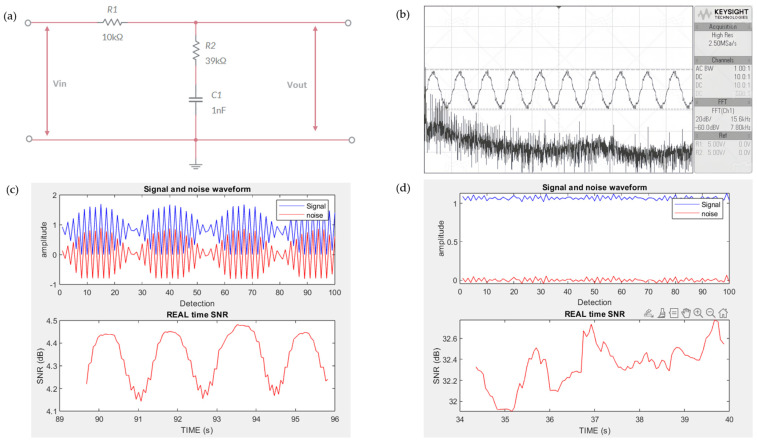
(**a**) A schematic diagram illustrating the design of the RC circuit for noise filtering. (**b**) The initial noise frequency of the TENG sensor was detected to be under 20 kHz using FFT. (**c**) The SNR value before connecting the TENG sensor to the RC circuit. (**d**). The SNR value after connecting the TENG sensor to the RC circuit, showing an improvement in signal quality.

To reduce the initial noise generated by the TENG sensors, fast Fourier transform (FFT) was employed to analyze the frequency spectrum of the noise signals. It was observed that, in the static initial state, the noise predominantly occurred below 20 kHz, as shown in Figure 3b. Based on this analysis, a resistor–capacitor (RC) filter circuit was designed to mitigate the noise effectively [18]. The goal was to filter out noise while preserving the meaningful TENG signals for further processing. The RC circuit was designed by using the following components: resistor R1 = 10 kΩ, capacitor C = 1 nF, and cutoff frequency Fc =20 kHz. The resistance of R2 was calculated to be 39 kΩ by using the cutoff frequency, as shown in Equation (1).

(1) $$Fc=\frac{1}{2\pi (\frac{R1\times R2}{R1+R2})C}$$

Figure 3a shows the RC circuit schematic diagram. After implementing the filter, the signal quality was verified using MATLAB to compare the signal-to-noise ratio (SNR) before and after the RC circuit was applied [19]. As shown in Figure 3c,d, the SNR increased from approximately 4.3 to 32.5, indicating a significant improvement in signal clarity because the signal was much stronger relative to the noise. This enhanced signal quality made it easier for signal processing algorithms to analyze the data, thus improving the overall system performance.

### 2.3. Signal Processing Logic for PLMS Detection

**Figure 4:**
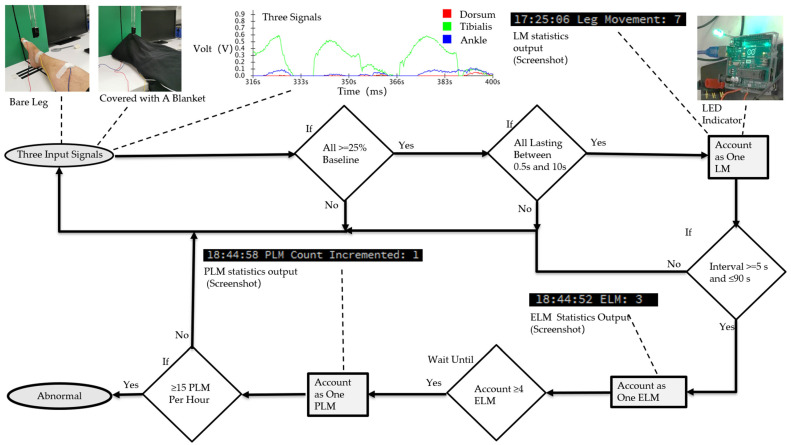
The logic flow of signal processing and judgment for PLMS detection. At the beginning of the test, the input signals from three synchronized sensors were recorded under various conditions (such as bare legs, covered with a blanket, one foot resting on the other, and rotating the foot left and right). Each movement was maintained for 1 s, and 10 trials were carried out to compute the mean value, with an error margin of 0.1 V. The baseline voltage was established at 0.15 V, and a threshold was set at 25% above the baseline, which is equal to 0.1875 V [5]. To visually represent the results, the graphs were plotted using the mean values of the signals, as displayed in Section 3. When all three sensors detected valid muscle activity lasting between 0.5 and 10 s simultaneously, the system classified this as a valid LM. If two consecutive LMs occurred with an interval between 5 and 90 s, this was considered a valid ELM. Once four ELMs were detected, it was classified as a PLMS. The system further monitored the occurrence of PLMS over a one-hour period. If 15 or more PLMS were detected, it was considered abnormal.

The experimental results show that relying on a single sensor signal can lead to inaccurate judgments due to external factors, such as one foot resting on the other or turning the foot left and right, which can generate false signals. However, simultaneous detection from three strategically placed sensors significantly improved the accuracy of PLMS detection by mitigating the influence of external factors. In this study, leg movement (LM) was defined as muscle activity above the baseline that lasted between 0.5 and 10 s. LM inter-movement intervals between 5 and 90 s were counted as episodes of leg movement (ELMs). PLMS consists of four or more consecutive ELMs [5]. PLMS is considered abnormal if it occurs more than 15 times per hour [4]. The logical flow of the PLMS signal processing and judgment for PLMS detection is shown in Figure 4.

To conduct the simulation test, an adult male participated in synchronized measurements on his right leg using three sensors under two distinct conditions: bare legs and covered with a blanket. For clarity, the activation of an LED light signified the occurrence of a valid leg movement (LM).

## 3. Results and Discussion

### 3.1. Single- and Multi-Sensor Performance Under Bare-Leg Conditions

**Figure 5:**
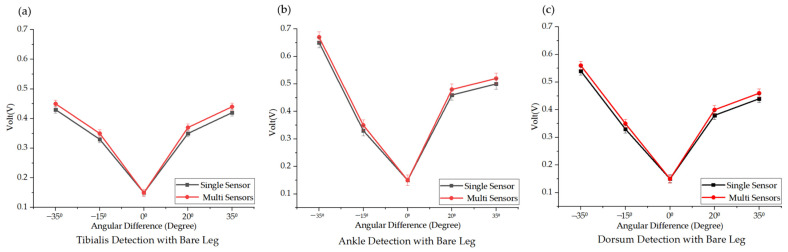
The experimental results under bare-leg conditions and comparison of the signal detection at the (**a**) tibialis anterior, (**b**) ankle position, and (**c**) dorsum of foot using a single sensor versus three synchronized sensors. Across all positions, the signals captured by the multi-sensor configuration closely match those obtained from the single sensor. This demonstrates the reliability of the detection capability of the multi-sensor approach, particularly for monitoring subtle movements associated with PLMS.

Traditional polysomnography (PSG) focuses on detecting signals from the tibialis anterior muscle under bare-leg conditions. In our study, to validate the effectiveness of our TENG approach in enhancing the detection of PLMS, we first tested the consistency between single-point and multi-point measurements. To simulate PLMS, dorsiflexion was performed at two fixed angles (90° and 110°) and plantar flexion at two angles (145° and 160°) under bare-leg conditions, utilizing both single and multiple sensors. The detected data are illustrated in Figure 5. The most significant muscle signal variations were observed at the ankle position, while changes at the tibialis location were comparatively weaker. Notably, greater dorsum movement amplitude correlated with larger muscle signal variations. This experiment verified consistency under traditional bare-leg conditions, confirming the reliability of the single and multi-TENG measurements according to the established standards.

### 3.2. Single- and Multi-Sensor Performance Under Blanket-Covered Conditions

**Figure 6:**
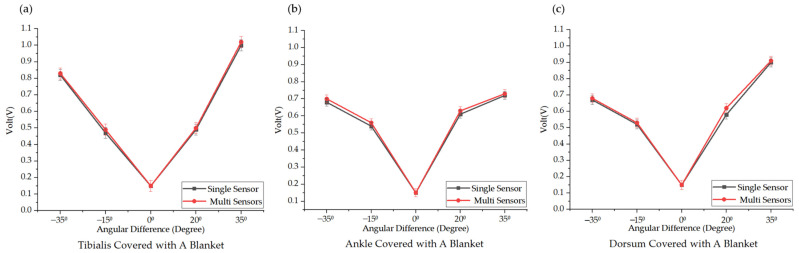
The experimental results under the blanket-covered conditions and comparison of signal detection at the (**a**) tibialis anterior, (**b**) ankle, and (**c**) dorsum of foot using a single sensor versus three synchronized sensors.

To break through the environmental limitations of traditional PSG, which requires bare-leg conditions, it was crucial to observe the detection performance of single- and multi-point TENGs when covered with a blanket. Being covered with a blanket is an important and common scenario during sleep at home. We conducted validation tests to evaluate whether consistency was also maintained between the single-point and multi-point measurements under this condition. To simulate PLMS, dorsiflexion was performed at two fixed angles (90° and 110°) and plantar flexion at two angles (145° and 160°) under blanket-covered conditions, utilizing both single and multiple sensors. The detected data are illustrated in Figure 6. The muscle signals across all detection points showed increased intensity due to the triboelectric nature of the TENG sensors. This indicates that the triboelectric response was amplified by the increased friction between the skin and the blanket.

During dorsiflexion and plantar flexion, the friction between the blanket and skin further enhanced the signal amplitude. Notably, at the tibialis anterior, when the angular deviation exceeded 35° from the natural resting position, the recorded muscle signals were stronger than those detected at the ankle and dorsum. At the dorsum of the foot, with the same 35° angular change, the signal variation during plantar flexion was noticeably greater than during dorsiflexion, likely due to the larger contact area between the dorsum and the blanket during plantar flexion, which intensified the triboelectric effect. The comparison between single- and multiple-sensor setups once again showed consistency, as the multi-sensor setup effectively captured muscle signals without significant deviation from the data recorded by the single sensor. This experiment verified, under real external disturbance conditions, the consistency between the single- and multi-TENG measurements.

### 3.3. Multi-Sensor Performance Under Bare-Leg and Blanket-Covered Conditions

**Figure 7:**
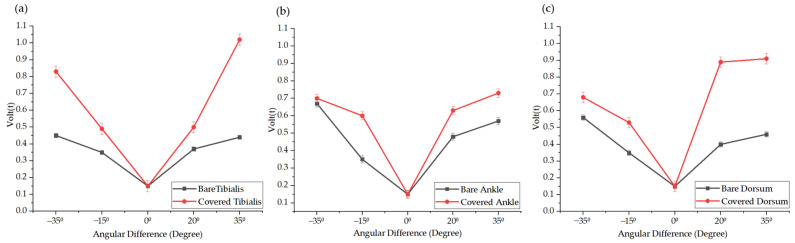
Comparison of the experimental comparison under bare-leg and blanket-covered conditions. Detection at the (**a**) tibialis anterior, (**b**) ankle, and (**c**) dorsum of the foot. Blanket friction plays a critical role in influencing sensor readings, particularly during larger movements. This phenomenon is more pronounced in home-based sleep monitoring environments, where users’ natural sleep postures may vary, and external influences, such as blankets, can alter sensor readings. Proper consideration of these factors and the inclusion of multi-point values are crucial for accurately interpreting PLMS signals.

In this experiment, we focused on using multiple TENG sensors to detect and compare signal variations between bare-leg and blanket-covered conditions. Specifically, we analyzed the signals from three monitoring points on the leg to evaluate the impact of different conditions on signal strength and variability. Under bare-leg conditions, the signal transmission was more direct, allowing for clearer charge generation and collection effects. In contrast, under the blanket-covered conditions, the friction between the blanket and the legs led to variations in the signals. These changes can significantly affect the accuracy of assessing PLMS. Figure 7 illustrates the comparison of muscle movements between the bare-leg and the blanket-covered conditions, revealing the significant impact of the blanket on the tibialis muscle, particularly during larger angle changes. When the dorsiflexion angle reached 35°, the signal detected from the tibialis muscle under the blanket condition was approximately double that under the bare-leg conditions. Similarly, at a plantar flexion angle of 35°, the signal was about 2.3 times greater under the blanket. For the ankle, the signals at all four angles increased under the blanket-covered conditions. Notably, when the dorsiflexion angle was at 35°, the change in the ankle signal was minimal in both conditions, with only a 5% increase under the blanket. However, at a dorsiflexion angle of 15°, the signal from the ankle under the blanket-covered conditions was approximately double that of the bare-leg conditions, which may have been due to increased friction between the ankle and the blanket at smaller angles of dorsiflexion. Regarding the dorsum, the blanket significantly amplified the signals during plantar flexion. When the dorsiflexion angle reached 20°, the signal from the dorsum under the blanket-covered conditions was about 2.25 times greater than that under the bare-leg conditions, and at 35°, it was approximately double.

These variations in signal intensity can be attributed to friction, indicating that more frequent or direct contact between the skin and the blanket during movement enhanced the triboelectric effects. The friction between the blanket and the leg resulted in significant signal changes at different leg positions, raising concerns about the accuracy of assessing PLMS based solely on readings from a single sensor under external disturbances. Values from multiple sensing locations provide a basis for a comprehensive evaluation of PLMS.

### 3.4. One Foot Resting on Top of the Other, and Turning Foot Left and Right Under Blanket-Covered and Bare-Leg Conditions

**Figure 8:**
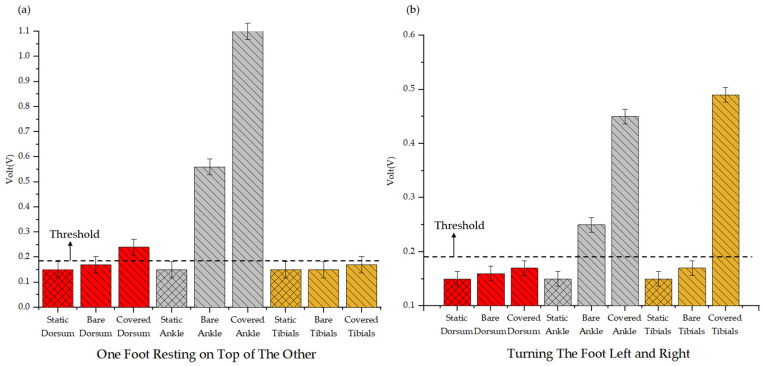
Signal variations with (**a**) one foot resting on top of the other and (**b**) left-to-right foot-turning movements.

In addition to validating detection capabilities under blanket-covered conditions, our study further investigated whether a multi-sensor approach can enhance the accuracy of PLMS assessments in non-PLMS activities. Previous research has usually simulated two scenarios to verify the occurrence of false signals. The first scenario involves one leg resting naturally while the other foot is placed on top of it. The second scenario involves the natural left-to-right turning motion of the foot. These conditions were tested under both the bare-leg (see Video S1—Bare legs) and blanket-covered (see Video S2—Covered with a blanket) setups. The measurement results are illustrated in Figure 8. However, neither of these scenarios fall under the classification of PLMS [3].

Using only a single sensor poses significant challenges in accurately distinguishing these non-PLMS activities from true PLMS. The signals generated by these non-PLMS activities can easily be misinterpreted as PLMS events. As shown in Figure 8a, when one foot was resting on top of the other under the blanket-covered conditions, there was no significant change in the tibialis signal, with detected values remaining below the threshold. In contrast, both the ankle and dorsum regions showed noticeable signal fluctuations above the threshold, indicating muscle activity. This effect was particularly pronounced in the ankle area, where triboelectric signals were elevated due to the contact between the legs. Under the bare-leg conditions, the dorsum and tibialis regions also detected values below the threshold, while the ankle area displays a clear signal change. In Figure 8b, when the foot naturally turned left and right under the blanket-covered conditions, there was no significant change in the dorsum signal, with detected values remaining below the threshold. However, both the ankle and tibialis sensors recorded noticeable changes in the muscle signals above the threshold, particularly under the blanket-covered scenario. This suggests that the blanket increased friction during foot rotation, thereby amplifying the signals. Under the bare-leg conditions, the dorsum and tibialis regions exhibited detected values below the threshold, while the ankle area demonstrated a distinct signal change above the threshold.

The following are the advantages of multi-sensor detection for PLMS assessment compared to single-sensor approaches: By combining data from multiple sensors, it becomes easier to distinguish between true PLMS and non-PLMS movements, significantly reducing the risk of misinterpretation. While a single sensor can provide valuable insights, it is highly susceptible to interference from external factors that may mimic PLMS signals. For example, friction from blankets or incidental foot contact can generate signals resembling PLMS events, leading to false positives and misclassifications. If only a single sensor is employed, these natural movements may be misclassified, as it may fail to differentiate between genuine PLMS and external artifacts. In contrast, employing multiple sensors strategically placed on different areas of the leg offers a more accurate solution. By analyzing muscle activity from various locations, this approach provides a comprehensive view of movement patterns, enabling more accurate detection of true PLMS events. The correlation between the signals from different sensors serves as a reliable indicator of whether a movement is legitimate PLMS or an artifact caused by external influences. The inclusion of data showing signal fluctuations detected by multiple sensors—even when one sensor showed no significant change—reinforces the argument for the necessity of multi-sensor configurations in reducing false positives and improving overall detection precision. This study shows the advantages of multi-sensor setups in improving the accuracy of assessing PLMS, particularly in complex movement scenarios where single-sensor data may lead to erroneous interpretations.

## 4. Conclusions

This research introduces several innovative aspects by using three key detection positions for TENG sensors—specifically, the dorsum, ankle, and anterior tibialis. Single-point and multi-point synchronous detection methods were employed to effectively identify periodic limb movements of sleep (PLMS). Initially, an RC circuit design was implemented to reduce noise in the TENG signals. The findings show that the TENG sensors can produce reliable detection results. The experimental results reveal a high degree of consistency between the single-point and multi-point detection outcomes, further confirming the effectiveness of the multi-sensor approach in mitigating signal interference. By synchronously capturing signals from multiple key joint positions, it shows that the multi-sensor method can effectively differentiate between genuine signals and the artifacts generated by a single sensor in the presence of external noise. Furthermore, when combined with signal processing logic for PLMS detection, our system enabled a more accurate assessment of PLMS occurrences. This multi-point detection system holds great promise for facilitating long-term, low-cost PLMS monitoring within home environments and establishes a solid foundation for the optimization and wider application of PLMS detection systems in the future.

## Data Availability

The data presented in this study are available upon request from the corresponding author. The data are not publicly available due to restrictions (privacy).

## Supplementary Materials

The following supporting information can be downloaded at: https://www.mdpi.com/article/10.3390/bios14110532/s1, Video S1: Bare legs; Video S2: Covered with a blanket.
